# 

**Published:** 1887-07

**Authors:** 


### CONTENTS.

### COMMUNICATIONS.

The importance of a Liberal Professional Education in the Practice of Dental and Oral Surgery........ 317
Status of Dentistry........ 327
Erythroxylon Coca and its Alkaloid—Cocaine........ 331
Preparation of Tissues for Examination with the Microscope........ 336

### SELECTIONS.

Right Upper Canine Tooth in the Left Orbit........ 342
Salivary Fistula Cured by the Galvano-Cautery........ 343
Action of Saline Purgatives........ 344
Important Metallurgical Discoveries of a Physician........ 344
Food for Reflection........ 345
The Advertisements of Religious Newspapers........ 348
Peroxide of Hydrogen........ 349
Pure Chromic Acid........ 349
College Commencement Exercises........ 351
SALMAGUNDI........ 359

### EDITORIAL.

The American Medical Association........ 361
International Medical Congress........ 363
American Dental Association Meeting........ 364
A New Syringe........ 365
Rates to Niagara Falls........ 365
Conditions of Membership in the Ninth International Medical Congress........ 366
The American Dental Association........ 367
The Southern Dental Association........ 368
Association of the Boards of Dental Examiners........ 368
National Association of Dental Examiners........ 368

